# Outcome measures in chronic obstructive pulmonary disease (COPD): strengths and limitations

**DOI:** 10.1186/1465-9921-11-79

**Published:** 2010-06-17

**Authors:** Thomas Glaab, Claus Vogelmeier, Roland Buhl

**Affiliations:** 1 Pulmonary Department, University Hospital, Johannes Gutenberg-University, Mainz, Germany; 2 Department of Pneumology, University Hospital Giessen and Marburg, Marburg, Germany

## Abstract

Current methods for assessing clinical outcomes in COPD mainly rely on physiological tests combined with the use of questionnaires. The present review considers commonly used outcome measures such as lung function, health status, exercise capacity and physical activity, dyspnoea, exacerbations, the multi-dimensional BODE score, and mortality. Based on current published data, we provide a concise overview of the principles, strengths and weaknesses, and discuss open questions related to each methodology. Reviewed is the current set of markers for measuring clinically relevant outcomes with particular emphasis on their limitations and opportunities that should be recognized when assessing and interpreting their use in clinical trials of COPD.

## Introduction

**Figure 1:**
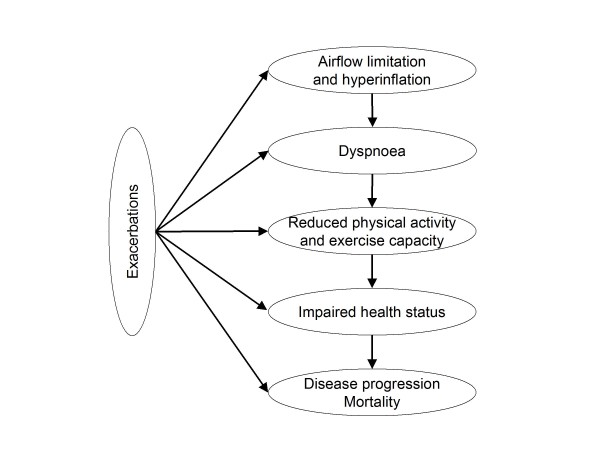
**Outcome measures relevant for the evaluation of COPD management**.

Chronic obstructive pulmonary disease (COPD) is a heterogeneous, multi-component disease associated with significant clinical burden. Though the presence of airflow limitation is well recognised as the pathophysiological basis, COPD as a complex disorder requires a multifaceted approach with regard to clinical assessment and response to therapy. This has prompted an intense search for clinical trial endpoints that may adequately reflect the success or failure of treatment. Current methods for assessing COPD progression mainly rely on lung function tests with a particular focus on forced expiratory volume in 1 second (FEV_1_). However, clinical and patient-reported outcome measures such as dyspnoea, exercise capacity, physical activity, exacerbations, health status and mortality have been recognized and applied as an essential part of the clinical assessment of COPD beyond FEV_1 _measurements [1,2] (figure 1).

In recent years, a profound analysis of available outcomes and markers has been provided by the scientific community [3,4]. The objective of this review is to provide a concise overview of the feasibility, strengths and limitations of major outcome measures commonly applied in current COPD trials.

### Lung function: forced expiratory volume in 1 second (FEV_1_)

#### Relevance

It is well established that patients with COPD lose lung function at a steeper rate than subjects without COPD. Post-bronchodilator forced expiratory volume (FEV_1_) is the single most important marker to determine severity and treatment algorithms in COPD. The decline of FEV_1 _over time has been traditionally used to indicate disease progression.

#### Measures

The diagnosis, staging and treatment of COPD in current guidelines is based on the fixed ratio of FEV_1_/FVC (forced vital capacity) and the percentage predicted FEV_1 _value.

The methodology for measuring forced expiratory maneuvers by spirometry has been standardized by ATS/ERS [5]. Specific training to yield reproducible and reliable results is mandatory.

#### Strengths

•FEV_1 _and FVC measurements are highly reproducible if performed adequately.

•Reduction in lung function is a risk factor for all cause and cardiovascular mortality [6-8], and impaired health status [9].

•Spirometry supports confirmatory detection of early stages of COPD when respiratory symptoms are often absent, thus creating the opportunity of early intervention [10].

#### Limitations

•FEV_1 _measurements are based on an artificial manoeuvre and do not always correlate with clinically relevant outcomes such as dyspnoea, health status, exercise capacity, or exacerbations [4,11].

•Patients with similar FEV_1 _may represent different underlying phenotypes.

•Reference equations for lung function by European Community for Coal and Steel are disputed and limited in predicting lung function in the general population [12].

•Changes in lung volumes can occur without concomitant changes in FEV_1 _and are more closely related than FEV_1 _changes to exercise performance [13].

•No minimal important difference (MID) has been established yet. It was suggested that an appropriate range of values for the MID for FEV_1 _might be 100-140 mL [4] but the MID for FEV_1 _remains poorly defined for COPD [14].

#### Open Questions and Outlook

FEV_1_, while a crucial marker, is far from being the only measure to comprehensively characterize patients with COPD. Additional outcome measures are usually needed to assess the clinical benefit of therapeutic agents. The relationships between changes in airway structure and measures of lung function require further investigation.

### Lung volumes

#### Relevance

Changes in absolute lung volumes can occur in COPD patients even in the absence of FEV_1 _changes. Progressive hyperinflation due to airflow limitation and loss of lung elastic recoil not only increases the work required during inspiration but also profoundly decreases the ventilatory reserve and increases the sense of effort and dyspnoea [15].

The assessement of absolute lung volumes has been standardized but is technically more demanding than simple spirometry. Specific training to yield reproducible and reliable results is essential.

#### Measures

Static lung hyperinflation and its increase during exercise (dynamic hyperinflation) are measured as elevations of total lung capacity (TLC), functional residual capacity (FRC), residual volume (RV) and as a decrease in inspiratory capacity (IC). The variability of lung volume measurements has been reviewed elsewhere [16].

#### Strengths

•Indices of dynamic hyperinflation correlate better than FEV_1 _with activity limitation and exertional dyspnoea [13,15] and pharmacological and surgical lung volume reduction have been associated with improvements in exercise performance and dyspnoea [17,18].

•A severely reduced IC/TLC ratio with a threshold value of 25% has been shown to predict mortality in COPD patients [19].

#### Limitations

•Body plethysmography remains the gold standard for the measurement of lung volumes such as TLC, FRC and RV. Spirometrically derived assessments of lung hyperinflation are more difficult to interpret in the absence of simultaneous bodyplethysmographic volume measurements to rule out a concomitant restrictive ventilatory disorder [15].

•The reproducibility of FRC, IC and RV in absolute values has yet to be demonstrated. Measurement of IC alone is not a reliable marker of lung hyperinflation and does not consistently reflect changes in FRC or TLC [15].

•Neither a standardized classification for the assessment of severity of hyperinflation nor a MID have been established yet. In practice, values of RV, TLC and FRC exceeding 120-130% of the predicted value are regarded to be clinically relevant, but these cut-offs are not validated.

•The natural course of dynamic hyperinflation in COPD is unknown and seems likely to be highly variable among COPD patients [15].

#### Open Questions and Outlook

In the absence of any consensus on the definition and/or severity of hyperinflation, it has been proposed that hyperinflation - preferentially expressed as % predicted- should be specified in terms of the volume compartment referred to and the measuring method used [12]. So far, there have been no studies aimed at exploring the longitudinal course of dynamic hyperinflation and its impact on the course of the disease in COPD patients.

### Exercise Capacity and Physical Activity

#### Relevance

Reduced exercise capacity is considered to be a consequence of airflow obstruction, primarily because of dynamic hyperinflation occurring during exercise. Reduced physical activity of patients is a result of COPD, but at the same time promotes worsening and progression of the disease [20].

#### Measures

There are different approaches to determine the exercise capacity or activity levels of COPD patients (table 1): Higher exercise tolerance measured via laboratory or field tests can be translated to higher levels of activity. In addition, physical activity during daily life can be assessed directly by measuring energy expenditure or by mechanical assessment of movement.

**Table 1:** Different methods to determine exercise capacity or activity levels in COPD

	Measure	Reference
field tests to determine exercise capacity	6-Minute Walk Test	[21]
	Shuttle Walk Test	[22,23]

laboratory tests to assess exercise capacity	bicycle ergometer	[24]
	treadmill	[25]
assessment of activity levels	accelerometer	[26]

### Exercise Capacity

#### 6-Minute Walk Test (6MWT)

Measurement of the distance walked during a 6-minute period on a level surface [21]. The principal outcome of this self-paced test is the distance covered. The MID is estimated to be 54-80 meters [27].

#### Strengths

•6MWT is relatively simple to perform and well tolerated.

•6MWT reflects everyday life-like activity.

•6MWT is validated and standardized [28].

•The test results correlate with lung function, health status, and maximal VO_2 _[29], and have shown to be predictive for mortality [30].

#### Limitations

•There are many sources of variability, e.g. patient's motivation, weight, height, age, sex, co-morbidities, and day-to-day variability [28].

•6MWT was significantly reduced only in COPD patients with GOLD stages III and IV [26,31].

•Assessment of the 6MWT is associated with spatial requirements and is personnel- and time-consuming.

•Standards of 6MWT are not always realisable. This might influence the results, e.g. shorter corridors reduce the distance covered because of time-consuming change in direction.

•Learning effect: Walking distance is up to 17% higher for a second test performed a day later [28].

#### Shuttle Walk Test (SWT)

There are two forms of assessment: In the Incremental Shuttle Walk Test, walking speed is set by the frequency of an acoustic signal. The frequency increases progressively until patients can no longer pick up the pace. The principal outcome is the distance covered. The MID is estimated to be 47.5 meters [32].

The Endurance Shuttle Walk Test has been developed to determine sub-maximal exercise capacity with the acoustic signal frequency being constant throughout the walk [23]. The principal outcome is the duration of exercise. No MID has yet been described.

#### Strengths

•SWT is relatively simple to perform and well-tolerated.

•Learning effects are minimal.

•Walking pace is externally controlled.

#### Limitations

•Instructions for SWT are time consuming.

•The test is less extensively validated than the 6MWT. Solid evidence for validity still has to be provided [27,33].

•SWT does not reflect common daily activities that require endurance and pacing.

#### Ergometry

To evaluate the exercise response, bicycle-ergometer or treadmill are commonly used in two different test modes. In incremental-workload tests, work-rate is increased progressively as a mild continuous ramp under computer control with the principal outcome being the distance covered. Alternatively, constant-workload tests have been performed at sub-maximal levels of exercise intensity which is typically set between 75% and 85% of the maximum workload during incremental tests [20]. The principal outcome is the duration of workload.

Reasons for break-off, e.g. leg discomfort vs. breathlessness, provide additional insights [34].

#### Strengths

•Standardized protocols are available [35].

•Treadmill walking reflects an activity of daily living.

•Cycle ergometer is less prone to introduce movement or noise artefacts into measurements than treadmill, and electrocardiogram and blood pressure are generally easier to measure [35].

•Additional physiological and clinical variables, such as peak O_2 _uptake, CO_2 _output, minute ventilation, heart rate, dyspnoea, and leg discomfort can be determined in parallel.

#### Limitations

•The workload not only depends on speed and inclination of the treadmill but also on the weight of the subject and pacing strategy. Body weight has much less effect on bicycle ergometry performance [30].

•Cycling is less closely related to the patient's activities of daily living.

•Resources: Ergometers are relatively expensive, treadmills require much space.

•No MID has been established yet.

### Physical Activity

#### Sensors for physical activity

The methods that are available to quantify physical activity in daily life include direct observation, assessment of energy expenditure, and the use of physical activity questionnaires or motion sensors. In particular, motion sensors are practical tools for clinical trials or practice. Accelerometers are electronic devices that record energy expenditure or mechanically assess movement. The devices are usually worn on patients' arm or waist. Accelerometers read out stored data as movement intensity and as quantity and can also provide data on body posture.

#### Strengths

•Accelerometers generate objective data by determination of quantity and intensity of body movements.

•Significant limitations of physical activity can already be detected in patients with moderate COPD (GOLD stage II) [31].

#### Limitations

•Solid evidence for reliability, validity and responsiveness for different types of accelerometers still has to be provided [26,31].

•Some activity sensors are poorly accepted by patients [36].

•Variability in sensitivity among accelerometers of a given model has been detected [37].

•Accelerometers may be sensitive to artefacts like car vibrations [26].

•Activity sensors may actually fail to accurately capture the inactive life style of patients with COPD [38].

•Physical activity patterns vary from day to day and between week-days and weekend due to the patient's health, or external factors [31]. In long-term studies, another source of variability may be seasonal climate changes, hours of daylight and weather [38].

•Observation bias: a greater level of activity may be induced during the measurement period that results in overestimation of the activity [39]. On the other hand, underreporting bias may evolve from poor compliance [26,39].

•No MID has been established yet.

#### Open questions and outlook

Exercise capacity is an important clinical outcome in interventional trials of COPD, but it is still debatable what is the most valid, reliable, and responsive measurement of changes within subjects.

Physical activity may become a key outcome measure not only in clinical trials of COPD, but also in rehabilitation programs and for patients' self-management. Even though the technical assessment of physical activity is improving rapidly, not all new techniques have been developed to the point where their clinical utility has been validated.

Little is known about the agreement of exercise capacity as measured using different methods. Therefore, indirect comparisons of treatment effects on exercise capacity are obscured by different methods of assessment applied in various trials.

### Dyspnoea

#### Relevance

For patients with COPD, dyspnoea is the most frequent complaint for which they seek medical attention. However, dyspnoea is a subjective measure that poorly correlates with objective assessments of lung function, exercise capacity, and other outcomes [1].

#### Measures

Different approaches have been used to measure dyspnoea in clinical trials, amongst which the BDI/TDI, Borg-Scale, and MRC are applied most often (table 2).

**Table 2:** Dyspnoea measurement scales

	Type of scale	Type of stimulus	Items	Administration
BDI/TDI	multi-dimensional	everyday activities	8/9	interview
MRC-Scale	uni-dimensional	everyday activities	1	self-administered by patient
Borg-Scale	uni-dimensional	under exertion	1	self-administered by patient

BDI: Baseline Dyspnoea Index; TDI: Transition Dyspnoea Index; MRC: Medical Research Council.

#### Baseline Dyspnoea Index/Transition Dyspnoea Index (BDI/TDI)

The BDI and TDI represent one of the most commonly applied instruments for dyspnoea rating in clinical trials, describing symptoms at a single point in time (e.g., baseline (BDI)), and measuring changes in breathlessness from this baseline state over time (TDI) [40].

BDI and TDI ratings are obtained in the course of an interview conducted by an experienced observer, who asks open-ended questions about the patient's experience of breathlessness during everyday activities, which are then translated into numerical values.

#### Strengths

•BDI and TDI ratings provide multi-dimensional measurements of breathlessness (functional impairment, magnitude of task, and magnitude of effort) related to activities of daily living.

•A MID with a difference of 1 unit for the mean total score being considered clinically meaningful is available though it is mainly based on retrospective data analysis [41,42].

#### Limitations

•Interviewer bias: Neither interviewer questions nor the translation of patients' answers to ratings are standardized, enforcing thorough interviewer training.

•Recall bias: The patient has to recall baseline state (BDI) in order to answer questions regarding the TDI.

•Assessment bias: Interviewer blinding to patients' clinical status is necessary to prevent assessment bias.

#### Medical Research Council (MRC) Scale

The MRC dyspnoea scale was developed as a simple and standardised method of categorising disability in COPD [4].

The patient selects a grade on the self-applied 5-point instrument that describes everyday situations or activity levels provoking breathlessness and impairment. A MID has not been established.

#### Strengths

•The method has been widely used in the past [43-46].

#### Limitations

•A possible underestimation bias due to avoidance of exertion has to be taken into account [47].

•The MRC is relatively insensitive to change, e.g. due to therapeutic intervention [48,49].

•There are relatively scarce clinical data on validation, responsiveness, and sensitivity [43].

#### Borg-Scale (CR-10)

The CR-10 or Borg-Scale has been developed primarily as an objective tool to measure exertional dyspnoea in COPD patients [50,51]. Although the 10-point category ratio scale is easy to use, concise and detailed instructions for patients are indispensable for appropriate application [52]. Based on retrospective analysis, a MID for the Borg-Scale in the range of 1 unit has been discussed [4].

#### Open Questions and Outlook

More research is needed to optimize and validate questionnaire items including direct patient involvement in instrument generation to improve their utility in clinical trials. Little is known about the impact of concomitant disorders on outcomes, e.g. if disorders such as anxiety or depression influence perceived dyspnoea and - if so - to which extent those instruments applied today reflect that influence. Furthermore, studies are needed to show which of the existing methodologies, e.g. questions or word lists, should be preferred in the context of COPD.

### Health Status

#### Relevance

Health-status is considered one of the main patient-related outcomes in clinical trials. It is important to make a distinction between quality of life (QoL), which is unique to the individual, and health status measurement, which is a standardized quantification of the impact of disease [53].

#### Measures

Health-status as a concept of high complexity is assessed indirectly and requires the application of specially designed questionnaires (table 3).

**Table 3:** Health-status measurement instruments

	Instrument Type	Domains	Items	Administration
SGRQ	disease-specific	symptoms, activities, psychosocial impact	76	self-administered by the patient
CRQ	disease-specific	dyspnea, emotional function, fatigue, mastery	20	interview
SF-36	generic	physical and social function, mental health, energy/vitality, health perception, physical and mental role limitation, pain	36	self-administered by the patient

SGRQ: fSt. George's Respiratory Questionnaire; CRQ: Chronic Respiratory Disease Questionnaire; SF-36: Short Form 36.

#### St. George's Respiratory Questionnaire (SGRQ)

The SGRQ was originally developed to measure health status in patients with respiratory disease, e.g. COPD or asthma [54]. A COPD-specific version is available [55].

The SGRQ covers domains of symptoms (frequency and severity of respiratory symptoms), activity (effects on and adjustment of everyday activities), and psychosocial impact, from which a total score with a possible maximum of 100 points is calculated.

The MID was assessed by various methods. Changes of 2 to 8 points were considered clinically meaningful, with a value of 4 applied most often [56].

#### Strengths

•The SGRQ has been widely used in clinical trials as a secondary endpoint to assess the effects of treatment and management interventions on health status in COPD.

•It may be considered a quasi standard in clinical trials.

#### Limitations

•The instrument is time-consuming to implement and is therefore of limited applicability in day-to-day clinical practice.

•There is a trend bias due to non-poled questions (first possible answer is usually "yes" and indicates worse health-status) [57].

•The processing of missing answers is unsatisfactory. A missing answer is considered as if the patient had answered "no" (indicating better health-status) [57].

•SGRQ scores were shown to be influenced by subjects' sex, age, education, and by comorbidities [58].

•Suitability of MID for individual patients as opposed to patient group comparisons has yet to be shown.

•Linearity of differences between SGRQ values has not been shown, especially not in different stages of severity. Thus, it is unknown, whether a reduction in SGRQ total score by 4 points (e.g. from 44 to 40) represents a subjective improvement in health status equivalent to a reduction from 64 to 60.

•There is little published empiric evidence supporting the MID of four points [59].

#### Chronic Respiratory Disease Questionnaire (CRQ)

The CRQ measures physical-functional and emotional limitations due to chronic lung diseases including COPD [60]. It refers to activity-related dyspnoea with results covering dyspnoea, fatigue, emotion, and mastery. The questionnaire has primarily been applied in rehabilitation trials of COPD patients [61].

The patient is asked to recall the five most important activities that caused breathlessness over the past two weeks. A total score as well as individual subscale scores can be calculated. A difference of 0.5 for the mean domain scores is considered clinically meaningful [62].

#### Strengths and limitations

A distinctive property of this instrument is the patient-specific selection of five activities, which cause dyspnoea for the individual patient. This way the instrument adapts to the specific conditions of the patient and is sensitive to treatment. On the other hand, the instrument is less suitable for inter-individual comparisons, as it mirrors individual physical limitations. The questionnaire is not interchangeable with other disease-specific instruments and has not yet been shown to be responsive to long-term disease progression.

#### Medical Outcomes Study Short Form-36 (SF-36)

The SF-36 is a generic health survey [63]. The patient is asked to complete 36 items of the questionnaire. The instrument allows the patient to self-assess psychic, physical, and social aspects of his or her quality of life.

#### Strengths and limitations

SF-36 is the best-known questionnaire to measure health status. The instrument has been shown to be discriminative, responsive to long-term disease progression, easy to use, and has been validated in several languages. However, as a generic measure, it is considered less responsive than disease-specific instruments in COPD and is not consistently responsive to therapeutic effects. No MID has been established yet.

#### Open questions and outlook

Further development of user-friendly, inexpensive instruments to enable fast and easy health status assessment in clinical trials as well as in daily practice is clearly required. Ways to involve patients in questionnaire generation should be further explored. More information is needed on the time course of health-status alterations (e.g., induced by therapeutic intervention or secondary to COPD exacerbations) and on the utility and efficacy of health status instruments in less severe COPD.

### Exacerbations

#### Relevance

Exacerbations of COPD indicate clinical instability and progression of the disease and are associated with increased morbidity, deterioration of comorbidities, reduced health status, physical and physiologic deterioration and an increased risk of mortality [64,65]. The prevention or reduction of exacerbations thus constitutes a major treatment goal [1].

#### Measures

Verification by patient interview, healthcare databases or prospectively from diary cards. Endpoints: frequency of exacerbations, time to first exacerbation, severity and duration of exacerbations.

#### Strenghts

•The event-based approach considers the need for systemic corticosteroids and/or antibiotics or hospitalisation due to an exacerbation. This definition may be more robust and is relatively easy to record.

•The symptom-based definition of exacerbations considers individual patient's perception of clinical status.

#### Limitations

•There is no standardized definition of an exacerbation, making comparative evaluations of clinical study results difficult [1,66].

•The symptom- and event-based approach involves subjective and recall bias, particularly because patients often have a poor understanding of exacerbation symptoms, resulting in substantial underreporting of exacerbations [67].

•The definition by use of health care resources is health system specific and affected by many other factors (social support, comorbidities, baseline health status, clinical expert behaviour).

•Differential diagnoses to exacerbations such as pneumonia, heart failure, ischemic heart disease, pulmonary embolism have to be taken into account.

•Seasonal variations in exacerbation frequency usually require long-term studies of at least one year duration [4,68].

•No MID has been established yet [4].

#### Open questions and outlook

There is a clear need to standardize the evaluation of the onset, frequency, severity and duration of COPD exacerbations as well as to assess therapeutic effects on exacerbations in COPD. Given the potential clinical relevance of even single exacerbations it appears quite difficult to determine exactly what cut-off levels should be used in terms of MIDs.

In addition, more work is needed to develop simple feasible criteria for defining exacerbations in clinical practice and to analyse the multiple factors that contribute to decisions to assess the severity stage of exacerbations. In that context, the EXACT-PRO initiative began to develop and evaluate a novel patient-reported outcome tool to measure the rate, duration and severity of exacerbations of COPD [69].

### Multidimensional scoring systems - BODE

#### Relevance

So far the only multidimensional scoring system that has gained broader acceptance is the BODE index which has been developed as a prognostic marker for COPD patients in an attempt to integrate not only the respiratory but also the systemic expressions of COPD in a single grading system [70].

#### Measures

It comprises the four components nutritional state (**B**MI), airflow limitation (**O**bstruction; FEV_1_), breathlessness (MRC **D**yspnoea scale), and **E**xercise capacity (6MWD, distance walked in 6 min). Replacing the 6MWD with a component for exacerbation frequency (BODEx index) resulted in fully preserved power to predict the mortality risk in a prospective observational study, while expanding the BODE index with exacerbation frequency as a fifth component (e-BODE index) did not further improve its predictive power [71]. A truncated version of the BODE index has been presented in which the exercise component is omitted (BOD index) [72].

The validity of the BODE index as a prognostic marker to predict mortality in COPD patients has recently been challenged by a study demonstrating that the risk of all-cause mortality over 3 years was considerably underestimated by the BODE index in a population of severe COPD patients, while on the contrary it was overestimated in another population with milder disease, indicating that important predictors may still be missing in this index [73]. Nevertheless, the BODE index has been used to assess therapeutic efficacy in interventional studies investigating effects of lung volume reduction surgery [74-76], pulmonary rehabilitation [77,78], and physical training [79], but so far not in pharmacological intervention trials.

#### Strengths

•The BODE index integrates different facets of COPD and the risks associated with significant comorbidities.

•It provides better power than that of its individual components (e.g., FEV_1_) to predict mortality and future exacerbations in patient populations with severe-to-very severe COPD [70,80].

•Its assessment is straightforward.

#### Limitations

•The BODE index has not primarily been developed to assess effects of therapeutic interventions and a MID has not yet been defined.

•The BODE index has been optimized to predict one-year mortality. The factors most critically affecting short-term survival might differ from those determining survival over a longer term. Thus, its suitability for assessment of patients with mild-to-moderate COPD is as yet less validated.

•The FEV_1 _categories in the airway obstruction component are not consistent with the current GOLD staging system

•No published experience with BODE index as a clinical outcome parameter in pharmacological intervention studies is currently available.

#### Open questions and outlook

More widespread application of the BODE index as an outcome parameter in clinical trials is currently hampered by the lack of experience in pharmacological intervention studies. Furthermore, its validity as a prognostic marker in a population of patients affected by mild-to-moderate COPD and its power to predict survival over longer periods of time as yet have to be proven.

### Mortality

#### Relevance

Long-term observations of large patient populations have shown an increased risk for all-cause mortality in COPD patients that rises proportionally to severity classes [6,8,81,82]. Mortality can be recorded as all-cause mortality and cause-specific mortality.

#### Strengths

•All-cause mortality is the most robust and reliable outcome of clinical trials in COPD and is relatively easy to follow-up [4,83,84].

#### Limitations

•Standardized methods to accurately define the cause of death (e.g. respiratory versus cardiovascular mortality) have not been established yet. Moreover, the careful analysis of the cause of death requires substantial effort.

•Retrospective mortality data may be confounded by inherent statistical bias [85,86], and even prospective studies are susceptible to bias due to missing follow-up of withdrawals [86,87].

•Adequately powered mortality trials require high patient numbers and extended study duration [3,84,88].

•It is as yet unclear, whether COPD-specific mortality is increased in patients with milder forms of COPD (GOLD stages I and II) [2,89].

•Mortality tends to be lower in participants of clinical trials than is found in routine clinical care [90].

#### Open Questions and Outlook

One important issue is the statistical approach to analyse the events of death. Intent-to-treat (ITT)-analyses, aiming for complete follow-up of deaths are recommended for unbiased comparison between treatment groups and should be used preferentially as shown in major trials [83,84,87].

For a confident, robust assessment, mortality should be the primary outcome of a prospective trial. Clinical trials evaluating death as a primary or secondary endpoint should have a data safety monitoring board and an independent adjudicating committee [3,4,91].

## Conclusion

The understanding of the merits and limitations of current methods for assessing physiological and clinical outcomes of COPD is crucial for the interpretation and design of clinical trials. Unfortunately, in contrast to monitoring lung function, there is no gold standard for measuring symptoms such as dyspnoea, health status, exercise capacity, physical activity, or exacerbations, since none of the available methods is optimal in all regards. Accordingly, no single outcome measure can be recommended for the assessment of treatment response in COPD. More research is needed to improve and simplify questionnaire-based markers or technologies to assess outcomes such as physical activity or health status in order to enable wider use in clinical trials as well as in primary care. A further step in that direction may be the recent development of a COPD assessment test [92].

Implementation of MIDs may also help to assess which changes of outcome markers can be considered clinically relevant. However, MIDs hardly reflect the heterogeneity, variability, and severity of COPD, as well as the numerous confounding factors contributing to the clinical presentation of the disease.

Further, no biomarkers have been established yet to reflect the inflammatory and destructive process in the lung or to indicate responsiveness to treatment. However, further research in this area is important as pulmonary biomarkers - whether physiological or biochemical - are urgently needed if clinical trials are to be shorter and more discriminating than at present.

Finally, comorbid conditions such as cardiovascular disease, anxiety and depressive disorders, lung cancer and osteoporosis are often observed in COPD patients and are likely to affect COPD outcomes. The impact of these conditions together with the influences of concomitant medication on COPD are variable and for many of them still uncertain; nevertheless, they may alter COPD phenotype, disease progression and survival, and responses to treatment. A systematic evaluation of comorbidities and co-medication should be considered as part of COPD management as they may influence the results of clinical outcome measures.

## Competing interests

TG was employee of Boehringer-Ingelheim at the time of manuscript submission. CV has given presentations at industry symposia sponsored by Altana, AstraZeneca, Aventis, Bayer, Boehringer-Ingelheim, Pfizer, GlaxoSmithKline, Merck Darmstadt, Talecris. He has also received consulting fees from Altana, AstraZeneca, Bayer, Boehringer-Ingelheim, Novartis, Pfizer, GlaxoSmithKline, Talecris. RB has received reimbursement for attending scientific conferences, and/or fees for speaking and/or consulting from AstraZeneca, Boehringer Ingelheim, Chiesi, GlaxoSmith Kline, Janssen-Cilag, Novartis, Nycomed, and Pfizer. The Pulmonary Department at Mainz University Hospital received financial compensation for services performed during participation in single- and multicenter clinical phase I-IV trials organized by various pharmaceutical companies.

## Authors' contributions

TG conceived of the review, drafted and coordinated the manuscript. CV and RB conceived of the review, critically discussed and helped to draft the manuscript. All authors read and approved the final manuscript.

## References

[B1] Rabe KF Hurd S Anzueto A Barnes PJ Buist SA Calverley P Fukuchi Y Jenkins C Rodriguez-Roisin R van Weel C Zielinski J Global strategy for the diagnosis, management, and prevention of chronic obstructive pulmonary disease: GOLD executive summary Am J Respir Crit Care Med 2007 176 532 555 10.1164/rccm.200703-456SO 17507545

[B2] Jones PW Agusti AG Outcomes and markers in the assessment of chronic obstructive pulmonary disease Eur Respir J 2006 27 822 832 10.1183/09031936.06.00145104 16585091

[B3] Gross N Outcome measures for COPD treatments: a critical evaluation COPD 2004 1 41 57 10.1081/COPD-120030413 16997738

[B4] Cazzola M MacNee W Martinez FJ Rabe KF Franciosi LG Barnes PJ Brusasco V Burge PS Calverley PMA Celli BR Jones PW Mahler DA Make B Miravitlles M Page CP Palange P Parr D Pistolesi M Rennard SI Rutten-van Mölken MP Stockley R Sullivan SD Wedzicha JA Wouters EF American Thoracic Society/European Respiratory Society Task Force on outcomes of COPD Outcomes for COPD pharmacological trials: from lung function to biomarkers Eur Respir J 2008 31 416 469 10.1183/09031936.00099306 18238951

[B5] Miller MR Hankinson J Brusasco V Burgos F Casaburi R Coates A Crapo R Enright P van der Grinten CP Gustafsson P Jensen R Johnson DC MacIntyre N McKay R Navajas D Pedersen OF Pellegrino R Viegi G Wanger J ATS/ERS Task Force: Standardisation of spirometry Eur Respir J 2005 26 319 338 10.1183/09031936.05.00034805 16055882

[B6] Sin DD Wu L Man SF The relationship between reduced lung function and cardiovascular mortality: a population-based study and a systematic review of the literature Chest 2005 127 1952 1959 10.1378/chest.127.6.1952 15947307

[B7] Ferrie JE Singh-Manoux A Kivimäki M Mindell J Breeze E Smith GD Shipley MJ Cardiorespiratory risk factors as predictors of 40-year mortality in women and men Heart 2009 95 1250 1257 10.1136/hrt.2008.164251 19389720 PMC2746941

[B8] Young RP Hopkins R Eaton TE Forced expiratory volume in one second: not just a lung function test but a marker of premature death from all causes Eur Respir J 2007 30 616 622 10.1183/09031936.00021707 17906084

[B9] Roche N Dalmay F Perez T Kuntz C Vergnenègre A Neukirch F Giordanella J-P Huchon G Impact of chronic airflow obstruction in a working population Eur Respir J 2008 31 1227 1233 10.1183/09031936.00089607 18216058

[B10] Akamatsu K Yamagata T Kida Y Tanaka H Ueda H Ichinose M Poor sensitivity of symptoms in early detection of COPD COPD 2008 5 269 273 10.1080/15412550802363303 18972274

[B11] Wise RA The value of forced expiratory volume in 1 second decline in the assessment of chronic obstructive pulmonary disease progression Am J Med 2006 119 4 11 10.1016/j.amjmed.2006.08.002 16996894

[B12] Pellegrino R Viegi G Brusasco V Crapo RO Burgos F Casaburi R Coates A van der Grinten CPM Gustafsson P Hankinson J Jensen R Johnson DC MacIntyre N McKay R Miller MR Navajas D Pedersen OF Wanger J Interpretative strategies for lung function tests Eur Respir J 2005 26 948 968 10.1183/09031936.05.00035205 16264058

[B13] O'Donnell DE Hyperinflation, dyspnea, and exercise intolerance in chronic obstructive pulmonary disease Proc Am Thorac Soc 2006 3 180 184 10.1513/pats.200508-093DO 16565429

[B14] Rabe KF Roflumilast for chronic obstructive pulmonary disease - author's reply Lancet 2005 366 1846 1847 10.1016/S0140-6736(05)67751-3 16310547

[B15] O'Donnell DE Laveneziana P Physiology and consequences of lung hyperinflation in COPD Eur Respir Rev 2006 15 61 67 10.1183/09059180.00010002

[B16] Hankinson JL Stocks J Peslin R Reproducibility of lung volume measurements Eur Respir J 1998 11 787 790 9596139

[B17] O'Donnell DE Is sustained pharmacologic lung volume reduction now possible in COPD? Chest 2006 129 501 503 10.1378/chest.129.3.501 16537841

[B18] Criner GJ Belt P Sternberg AL Mosenifar Z Make BJ Utz JP Sciurba F National Emphysema Treatment Trial Research Group. Effects of lung volume reduction surgery on gas exchange and breathing pattern during maximum exercise Chest 2009 135 1268 79 10.1378/chest.08-1625 19420196 PMC2818416

[B19] Casanova C Cote C de Torres JP Aguirre-Jaime A Marin JM Pinto-Plata V Celli BR Inspiratory-to-total lung capacity predicts mortality in patients with chronic obstructive pulmonary disease Am J Respir Crit Care Med 2005 171 591 597 10.1164/rccm.200407-867OC 15591470

[B20] Palange P Ward SA Carlsen K-H Casaburi R Gallagher CG Gosselink R O'Donnell DE Puente-Maestu L Schols AM Singh S Whipp BJ Recommendations on the use of exercise testing in clinical practice Eur Respir J 2007 29 185 209 10.1183/09031936.00046906 17197484

[B21] Butland RJA Pang J Gross ER Two-, six-, and 12-minute walk tests in respiratory disease BMJ 1982 284 1607 1608 10.1136/bmj.284.6329.1607 6805625 PMC1498516

[B22] Singh SJ Morgan MDL Scott S Walters D Hardman AE Development of a shuttle walk test of disability in patients with chronic airways obstruction Thorax 1992 47 1019 1024 10.1136/thx.47.12.1019 1494764 PMC1021093

[B23] Revill SM Morgan MDL Singh SJ Williams J Hardman AE The endurance shuttle walk: a new field test for the assessment of endurance capacity in chronic obstructive pulmonary disease Thorax 1999 54 213 222 10.1136/thx.54.3.213 10325896 PMC1745445

[B24] Whipp BJ Davis JA Torres F Wasserman K A test to determine the parameters of aerobic function during exercise J Appl Physiol 1981 50 217 221 6782055 10.1152/jappl.1981.50.1.217

[B25] Porszasz J Casaburi R Somfay A Woodhouse LJ Whipp BJ A treadmill ramp protocol using simultaneous changes in speed and grade Med Sci Sports Exerc 2003 35 1596 1603 10.1249/01.MSS.0000084593.56786.DA 12972882

[B26] Pitta F Troosters T Probst VS Spruit MA Decramer M Gosselink R Quantifying physical activity in daily life with questionnaires and motion sensors in COPD Eur Respir J 2006 27 1040 1055 16707399 10.1183/09031936.06.00064105

[B27] Wise RA Brown CD Minimal clinically important difference in the six-minute walk test and the incremental shuttle walk test COPD 2005 2 125 129 10.1081/COPD-200050527 17136972

[B28] ATS statement Guidelines for the six-minute walk test Am J Respir Crit Care Med 2002 166 111 117 12091180 10.1164/ajrccm.166.1.at1102

[B29] Brown CD Wise RA Field tests of exercise in COPD: The six-minute walk test and the shuttle walk test COPD 2007 4 217 223 10.1080/15412550701480125 17729065

[B30] Cote CG Casanova C Marín JM Lopez MV Pinto-Plata V De Oca MM Dordelly LJ Nekach H Celli BR Validation and comparison of reference equations for the 6-min walk distance test Eur Respir J 2008 31 571 578 10.1183/09031936.00104507 17989117

[B31] Watz H Waschki B Meyer T Magnussen H Physical activity in patients with COPD Eur Respir J 2009 33 262 272 10.1183/09031936.00024608 19010994

[B32] Singh SJ Jones PW Evans R Morgan MDL Minimum clinically important improvement for the incremental shuttle walking test Thorax 2008 63 775 777 10.1136/thx.2007.081208 18390634

[B33] Solway S Brooks D Lacasse Y Thomas S A qualitative systemic overview of the measurement properties of functional walk tests used in the cardiorespiratory domain Chest 2001 119 256 270 10.1378/chest.119.1.256 11157613

[B34] Murray JA Waterman LA Ward J Baird JC Mahler DA Perceptual and physiologic responses during treadmill and cycle exercise in patients with COPD Chest 2009 135 384 390 10.1378/chest.08-1258 18753470

[B35] American Thoracic Society ATS/ACCP Statement on cardiopulmonary exercise testing Am J Respir Crit Care Med 2003 167 211 277 10.1164/rccm.167.2.211 12524257

[B36] Fensli R Pedersen PE Gundersen T Hejlesen O Sensor acceptance model - measuring patient acceptance of wearable sensors Methods Inf Med 2008 47 89 95 18213434 10.3414/me9106

[B37] Morgan M Life in slow motion: quantifying physical activity in COPD Thorax 2008 63 663 664 10.1136/thx.2007.093492 18663063

[B38] Troosters T Physical activity monitoring: a new outcome facing many challenges, but yielding promising results COPD 2009 6 82 83 10.1080/15412550902806037 19378219

[B39] Casaburi R Activity Monitoring in assessing activities of daily living COPD 2007 4 251 255 10.1080/15412550701480158 17729069

[B40] Mahler DA Weinberg DH Wells CK Feinstein AR The measurement of dyspnea. Contents, interobserver agreement, and physiologic correlates of two new clinical indexes Chest 1984 85 751 758 10.1378/chest.85.6.751 6723384

[B41] Mahler DA Witek TJ The MCID of the transition dyspnea index is a total score of one unit COPD 2005 2 99 103 10.1081/COPD-200050666 17136969

[B42] Witek TJ Mahler DA Minimal important difference of the transition dyspnoea index in a multinational clinical trial Eur Respir J 2003 21 267 272 10.1183/09031936.03.00068503a 12608440

[B43] de Torres JP Pinto-Plata V Ingenito E Bagley P Gray A Berger R Celli B Power of outcome measurements to detect clinically significant changes in pulmonary rehabilitation of patients with COPD Chest 2002 121 1092 1098 10.1378/chest.121.4.1092 11948037

[B44] Watson L Vestbo J Postma DS Decramer M Rennard S Kiri VA Vermeire PA Soriano JB Gender differences in the management and experience of Chronic Obstructive Pulmonary Disease Respir Med 2004 98 1207 1213 10.1016/j.rmed.2004.05.004 15588042

[B45] van Wetering CR van Nooten FE Mol SJM Hoogendoorn M Rutten-van Mölken MPMH Schols AM Systemic impairment in relation to disease burden in patients with moderate COPD eligible for a lifestyle program. Findings from the INTERCOM trial Int J Chron Obstruct Pulmon Dis 2008 3 443 451 18990973 10.2147/copd.s2588 PMC2629991

[B46] Bourbeau J Ford G Zackon H Pinsky N Lee J Ruberto G Impact on patients' health status following early identification of a COPD exacerbation Eur Respir J 2007 30 907 913 10.1183/09031936.00166606 17715163

[B47] Rennard S Decramer M Calverley PM Pride NB Soriano JB Vermeire PA Vestbo J Impact of COPD in North America and Europe in 2000: subjects' perspective of Confronting COPD International Survey Eur Respir J 2002 20 799 805 10.1183/09031936.02.03242002 12412667

[B48] Mahler DA Mahler DA Measurement of dyspnea: clinical ratings In Dyspnea: Mechanisms, Measurement and Management 2005 2 New York: Taylor and Francis 147 164

[B49] Haughney J Gruffydd-Jones K Patient-centred outcomes in primary care management of COPD - what do recent clinical trial data tell us? Prim Care Resp J 2004 13 185 197 10.1016/j.pcrj.2004.06.006 PMC6750695 16701668

[B50] Borg G Psychophysical bases of perceived exertion Med Sci Sports Exerc 1982 14 377 381 7154893

[B51] Borg G Psychophysical scaling with applications in physical work and the perception of exertion Scand J Work Environ Health 1990 16 Suppl 1 55 58 2345867 10.5271/sjweh.1815

[B52] Mador MJ Rodis A Magalang UJ Reproducibility of Borg scale measurements of dyspnea during exercise in patients with COPD Chest 1995 107 1590 1597 10.1378/chest.107.6.1590 7781352

[B53] Jones PW Health status and the spiral of decline COPD 2009 6 59 63 10.1080/15412550802587943 19229709

[B54] Jones PW Quirk FH Baveystock CM Littlejohns P A self-complete measure of health status for chronic airflow limitation. The St. George's Respiratory Questionnaire Am Rev Respir Dis 1992 145 1321 1327 1595997 10.1164/ajrccm/145.6.1321

[B55] Meguro M Barley EA Spencer S Jones PW Development and Validation of an Improved, COPD-Specific Version of the St. George Respiratory Questionnaire Chest 2007 132 456 463 10.1378/chest.06-0702 17646240

[B56] Jones PW Interpreting thresholds for a clinically significant changes in health status in asthma and COPD Eur Respir J 2002 19 398 404 10.1183/09031936.02.00063702 11936514

[B57] Mühlig S Petermann F Illness specific data collection on quality of life of patients with asthma and chronic obstructive bronchitis Rehabilitation 1998 37 25 38 9789318

[B58] Ferrer M Villasante C Alonso J Sobradillo V Gabriel R Vilagut G Masa JF Viejo JL Jiménez-Ruiz CA Miravitlles M Interpretation of quality of life scores from the St George's Respiratory Questionnaire Eur Respir J 2002 19 405 413 10.1183/09031936.02.00213202 11936515

[B59] Schünemann HJ Griffith L Jaeschke R Goldstein R Stubbing D Guyatt GH Evaluation of the minimal important difference for the feeling thermometer and the St. George's Respiratory Questionnaire in patients with chronic airflow obstruction J Clin Epidemiol 2003 56 1170 1176 10.1016/S0895-4356(03)00115-X 14680667

[B60] Guyatt GH Berman LB Townsend M Pugsley SO Chambers LW A measure of quality of life for clinical trials in chronic lung disease Thorax 1987 42 773 778 10.1136/thx.42.10.773 3321537 PMC460950

[B61] Puhan MA Guyatt GH Goldstein R Mador J McKim D Stahl E Griffith L Schünemann HJ Relative responsiveness of the Chronic Respiratory Questionnaire, St. Georges Respiratory Questionnaire and four other health-related quality of life instruments for patients with chronic lung disease Respir Med 2007 101 308 316 10.1016/j.rmed.2006.04.023 16782320

[B62] Schünemann HJ Puhan M Goldstein R Jaeschke R Guyatt GH Measurement properties and interpretability of the Chronic respiratory disease questionnaire (CRQ) COPD 2005 2 81 89 10.1081/COPD-200050651 17136967

[B63] Ware JE Jr Gandek B Overview of the SF-36 Health Survey and the International Quality of Life Assessment (IQOLA) Project J Clin Epidemiol 1998 51 903 912 10.1016/S0895-4356(98)00081-X 9817107

[B64] Wedzicha JA Seemungal TA COPD exacerbations: defining their cause and prevention Lancet 2007 370 786 796 10.1016/S0140-6736(07)61382-8 17765528 PMC7134993

[B65] Anzueto A Sethi S Martinez FJ Exacerbations of chronic obstructive pulmonary disease Proc Am Thorac Soc 2007 4 554 564 10.1513/pats.200701-003FM 17878469

[B66] Pauwels R Calverley P Buist AS Rennard S Fukuchi Y Stahl E Löfdahl CG COPD exacerbations: the importance of a standard definition Respir Med 2004 98 99 107 10.1016/j.rmed.2003.09.001 14971871

[B67] Kessler R Ståhl E Vogelmeier C Haughney J Trudeau E Löfdahl CG Partridge MR Patient understanding, detection, and experience of COPD exacerbations: an observational, interview-based study Chest 2006 130 133 142 10.1378/chest.130.1.133 16840393

[B68] Burge S Wedzicha JA COPD exacerbations: definitions and classifications Eur Respir 2003 21 46s 53s 10.1183/09031936.03.00078002 12795331

[B69] Jones P Higenbottam T Quantifying of severity of exacerbations in chronic obstructive pulmonary disease: adaptations to the definition to allow quantification Proc Am Thorac Soc 2007 4 597 601 10.1513/pats.200707-115TH 18073389

[B70] Celli BR Cote CG Marin JM Casanova C Montes de Oca M Mendez RA Pinto Plata V Cabral HJ The Body-mass index, airflow obstruction, dyspnea, and exercise capacity index in chronic obstructive pulmonary disease N Engl J Med 2004 350 1005 1012 10.1056/NEJMoa021322 14999112

[B71] Soler-Cataluña JJ Martínez-García MÁ Sánchez LS Tordera MP Sánchez PR Severe exacerbations and BODE index: Two independent risk factors for death in male COPD patients Respir Med 2009 103 692 699 10.1016/j.rmed.2008.12.005 19131231

[B72] Celli BR Calverley PMA Rennard SI Wouters EFM Agusti A Anthonisen N MacNee W Jones P Pride N Rodriguez-Roisin R Rossi A Wanner A Proposal for a multidimensional staging system for chronic obstructive pulmonary disease Respir Med 2005 99 1546 1554 10.1016/j.rmed.2005.03.019 16291077

[B73] Puhan MA Garcia-Aymerich J Frey M ter Riet G Antó JM Agustí AG Gómez FP Rodríguez-Roisín R Moons KGM Kessels AG Held U Expansion of the prognostic assessment of patients with chronic obstructive pulmonary disease: the updated BODE index and the ADO index Lancet 2009 374 704 711 10.1016/S0140-6736(09)61301-5 19716962

[B74] Lederer DJ Thomashow BM Ginsburg ME Austin JH Bartels MN Yip CK Jellen PA Brogan FL Kawut SM Maxfield RA DiMango AM Simonelli PF Gorenstein LA Pearson GD Sonett JR Lung-volume reduction surgery for pulmonary emphysema: Improvement in body mass index, airflow obstruction, dyspnea, and exercise capacity index after 1 year J Thorac Cardiovasc Surg 2007 133 1434 1438 10.1016/j.jtcvs.2006.12.062 17532935

[B75] Pompeo E Mineo TC Two-year improvement in multidimensional body mass index, airflow obstruction, dyspnea, and exercise capacity index after nonresectional lung volume reduction surgery in awake patients Ann Thorac Surg 2007 84 1862 1869 10.1016/j.athoracsur.2007.07.007 18036900

[B76] Martinez FJ Han MK Andrei AC Wise R Murray S Curtis JL Sternberg A Criner G Gay SE Reilly J Make B Ries AL Sciurba F Weinmann G Mosenifar Z DeCamp M Fishman AP Celli BR National Emphysema Treatment Trial Research Group. Longitudinal change in the BODE index predicts mortality in severe emphysema Am J Respir Crit Care Med 2008 78 491 499 10.1164/rccm.200709-1383OC PMC2542428 18535255

[B77] Cote CG Celli BR Pulmonary rehabilitation and the BODE index in COPD Eur Respir J 2005 26 630 636 10.1183/09031936.05.00045505 16204593

[B78] Foglio K Bianchi L Bruletti G Porta R Vitacca M Balbi B Ambrosino N Seven-year time course of lung function, symptoms, health-related quality of life and exercise toleranc in COPD patients undergoing pulmonary rehabilitation programs Respir Med 2007 101 1961 1970 10.1016/j.rmed.2007.04.007 17531455

[B79] Nasis IG Vogiatzis I Stratakos G Athanasopoulos D Koutsoukou A Daskalakis A Spetsioti S Evangelodimou A Roussos C Zakynthoinos S Effects of interval-load versus constant-load training on the BODE index in COPD patients Respir Med 2009 103 1392 1398 10.1016/j.rmed.2009.03.003 19349153

[B80] Marin JM Carrizo SJ Casanova C Martinez-Camblor P Soriano JB Agusti AG Celli BR Prediction of risk of COPD exacerbations by the BODE index Respir Med 2009 103 373 378 10.1016/j.rmed.2008.10.004 19013781

[B81] Mannino DM Buist AS Petty TL Enright PL Redd SC Lung function and mortality in the United States: data from the First National Health and Nutriation Examination Survey follow up study Thorax 2003 58 388 393 10.1136/thorax.58.5.388 12728157 PMC1746680

[B82] Ekberg-Aronsson M Pehrsson K Nilsson JÅ Nilsson PM Löfdahl CG Mortality in GOLD stages of COPD and its dependence on symptoms of chronic bronchitis Respir Res 2005 6 98 10.1186/1465-9921-6-98 16120227 PMC1224873

[B83] Tashkin DP Celli B Senn S Burkhart D Kesten S Menjoge S Decramer M A 4-year trial for Tiotropium in chronic obstructive pulmonary disease N Engl J Med 2008 359 1543 1554 10.1056/NEJMoa0805800 18836213

[B84] Calverley PMA Anderson JA Celli B Ferguson GT Jenkins C Jones PW Yates JC Vestbo J Salmeterol and fluticasone propionate and survival in chronic obstructive pulmonary disease N Engl J Med 2007 356 775 789 10.1056/NEJMoa063070 17314337

[B85] Sin D Tu J Inhaled corticosteroids and the risk of mortality and re-admission in elderly patients with chronic obstructive pulmonary disease Am J Respir Crit Care med 2001 164 580 584 11520719 10.1164/ajrccm.164.4.2009033

[B86] Suissa S Effectiveness of inhaled corticosteroids in COPD, immortal time bias in observational studies Am J Respir Crit Care Med 2003 168 49 53 10.1164/rccm.200210-1231OC 12663327

[B87] Suissa S Barnes PJ Inhaled corticosteroids in COPD: the case against Eur Respir J 2009 34 13 16 10.1183/09031936.00190908 19567599

[B88] Anthonisen NR Skeans MA Wise RA Manfreda J Kanner RE Connett JE The effects of smoking cessation intervention on 14.5-year mortality Ann Int Med 2005 142 233 239 15710956 10.7326/0003-4819-142-4-200502150-00005

[B89] Sin DD Wu L Anderson JA Anthonisen NR Buist AS Burge PS Calverley PM Connett J.E Lindmark B Pauwels R.A Postma D.S Soriano J.B Szafranski W Vestbo J Inhaled corticosteroids and mortality in chronic obstructive pulmonary disease Thorax 2005 60 992 997 10.1136/thx.2005.045385 16227327 PMC1747271

[B90] Niewoehner DE Erbland ML Deupree RH Collins D Gross NJ Light RW Anderson P Morgan NA Effect of systemic glucocorticoids on exacerbations of chronic obstructive pulmonary disease N Engl J Med 1999 340 1941 1947 10.1056/NEJM199906243402502 10379017

[B91] Sin DD Anthonisen NR Soriano JB Agusti AG Mortality in COPD: role of co-morbidities Eur Respir J 2006 28 1245 1257 10.1183/09031936.00133805 17138679

[B92] Jones PW Harding G Berry P Wiklund I Chen WH Kline Leidy N Development and first validation of the COPD Assessment Test Eur Respir J 2009 34 648 654 10.1183/09031936.00102509 19720809

